# Review of Basic Research about Ossification of the Spinal Ligaments Focusing on Animal Models

**DOI:** 10.3390/jcm12051958

**Published:** 2023-03-01

**Authors:** Masato Ikuta, Takashi Kaito, Takahito Fujimori, Takayuki Kitahara, Takuya Furuichi, Masayuki Bun, Hiromasa Hirai, Yuichiro Ukon, Yuya Kanie, Shota Takenaka, Seiji Okada

**Affiliations:** Department of Orthopedic Surgery, Osaka University Graduate School of Medicine, 2-2 Yamadaoka, Suita 565-0871, Osaka, Japan

**Keywords:** ossification of the posterior longitudinal ligament (OPLL), ossification of the spinal ligaments (OSL), animal models

## Abstract

Ossification of the posterior longitudinal ligament (OPLL) is a heterotopic ossification that may cause spinal cord compression. With the recent development of computed tomography (CT) imaging, it is known that patients with OPLL often have complications related to ossification of other spinal ligaments, and OPLL is now considered part of ossification of the spinal ligaments (OSL). OSL is known to be a multifactorial disease with associated genetic and environmental factors, but its pathophysiology has not been clearly elucidated. To elucidate the pathophysiology of OSL and develop novel therapeutic strategies, clinically relevant and validated animal models are needed. In this review, we focus on animal models that have been reported to date and discuss their pathophysiology and clinical relevance. The purpose of this review is to summarize the usefulness and problems of existing animal models and to help further the development of basic research on OSL.

## 1. Introduction

Ossification of the posterior longitudinal ligament (OPLL) is a heterotopic ossification of ligaments that occurs primarily in the cervical spine. This hyperostic condition was recognized by rheumatologists or radiologists in 19th century. After a report of an autopsy case in 1960, the ossification of the posterior longitudinal ligament (OPLL) became known as one cause of myelopathy.

Patients with OPLL sometimes develop severe myelopathy, which may require surgical treatment. Although surgical treatment is effective, it is difficult to fully restore spinal cord function once impaired. Patients with myelopathy due to OPLL live with a variety of residual symptoms. Epidemiological studies have shown that the prevalence is higher in Japanese individuals, ranging from 1.9% to 4.3% compared to 0.1% to 1.3% in people of European descent [1]. Among other races, the figures are 4.8% of Asian Americans, 1.9% of Hispanic Americans, 2.1% of African Americans, and 3.2% of Native Americans. The average age of onset is reported to be over 60 years, and the disease occurs predominantly in males. Cervical OPLL accounts for 80% of the onset sites. Thoracic OPLL accounts for about 10%, and lumbar OPLL about 5%. On the other hand, ossification of the ligamentum flavum occurs more frequently in the thoracic spine [2,3].

Recently, whole-spine computed tomography (CT) imaging has been widely used to accurately assess ossification lesions and it has been found that patients with cervical OPLL also have a high rate of ossification of other spinal ligaments [4,5]. According to these reports, in patients with cervical OPLL, diffuse idiopathic hyperostosis (DISH) has been reported to occur in 47.8% of patients. Similarly, 64.6% of cervical OPLL patients were found to have a combination of ossification of the ligamentum flavum (OLF), 55.8% of the nuchal ligament (ONL), and 29% of the supra/interspinous ligament (OSIL) [6,7,8,9]. Thus, given the tendency of patients with OPLL to ossify spinal ligaments in various locations, OPLL has recently been considered a part of ossification of spinal ligaments (OSL).

Many reports suggest that OSL is a multifactorial disease with a mixture of genetic and environmental (non-genetic) factors. High prevalence has been reported in parents and siblings of patients with OPLL. HLA haplotype analysis showed that 53% of siblings with a matched double-stranded HLA haplotype developed OPLL, compared to 24% of siblings with a matched single-stranded HLA haplotype and 5% of those with no match [10,11]. Environmental factors such as diet, mechanical stress, and endocrine metabolic abnormalities have been reported. Several systemic hormones, including 1,25-dihydoxy vitamin D, parathyroid hormone (PTH), insulin, and leptin, are thought to be involved in the initiation and development of OSL. A study of Taiwanese subjects reported that a high-salt diet with high pickle intake and low daily meat intake were positively associated with the development of OPLL. A study of Japanese subjects reported that frequent consumption of chicken and soy foods was associated with a decreased risk of developing OPLL. In contrast, regular consumption of pickles was associated with an increased risk. Furthermore, vitamin A deficiency resulting from an unbalanced diet was reported to be associated with worse early-onset OPLL in men [12,13,14,15,16,17]. Histochemical studies of OPLL have shown the presence of several different phenotypic osteoblasts in ligament cells obtained from non-osteogenic sites. In addition, they exhibit high alkaline phosphatase (ALP) activity, increased parathyroid hormone- and prostaglandin E2-stimulated cAMP, and features of response to both calcification and 1,25-dihydroxycholecalciphenol. In the past, the administration of bisphosphonates has been attempted to prevent ectopic ossification, but with unsatisfactory results. Currently, no effective treatment has been established other than symptomatic surgical therapy [18]. However, as mentioned above, surgery also relieves pressure on the spinal cord and does not fundamentally prevent ossification progression. Thus, the underlying pathogenesis of OSL remains to be elucidated, and early elucidation of the pathogenesis of OSL is desirable. Animal models based on the pathogenesis of OSL can be helpful to elucidate the pathogenesis and develop effective therapies.

The following two topics are summarized. The first is the histology of human OSL. We investigated the process by which ligaments undergo ossification. The second describes the major animal models reported to date and the pathogenesis of OSL based on them. We will also discuss prospects for future research based on these findings.

## 2. Histology of Human OSL and Hypothesis of Ossification Pathogenesis

### 2.1. Histology of Human OPLL

Posterior longitudinal ligament (PLL) is a fibrous connective tissue that connects the vertebral body to other ones. PLL is located in the spinal canal and covers the posterior surfaces of the vertebral bodies, and consists of the superficial layer and the deep layer. These ligaments attach to the vertebral body through the enthesis. The human spinal ligament attachments are composed of four areas: the bone area, the calcified cartilage area, the fibrocartilage area, and the ligamentous area. The calcified cartilage area contains calcium deposits in addition to the cartilage matrix, such as type II and type X collagen and proteoglycans. The fibrocartilage area consists of increased type II collagen and proteoglycans and round chondrocytes. The ligamentous area consists of collagen fibers and spindle-shaped fibroblasts [19]. 

The ossification front of OPLL presents a similar structure. Prominent wavy and irregular calcified cartilage areas are observed in the ossification front, with numerous hypertrophic chondrocytes. Fibrocartilage areas near the ossification front contain bone morphogenetic protein-2 (BMP-2)-positive cells. Fibrous structures in the ligament areas show marked degeneration and disruption in the calcified cartilage area. Terminal transferase dUTP nick-end labeling (TUNEL)-positive hypertrophic chondrocytes, which indicate apoptosis, are observed mainly in the fibrocartilage area near the calcified cartilage area [20]. Chondrocytes proliferating in the fibrocartilage area are strongly positive for SRY-type HMG box9 (Sox9), a transcription factor for chondrogenic differentiation, and cluster of differentiation 90 (CD90), a mesenchymal stem cell marker. Runt-related transcription factor 2 (Runx2), a transcription factor for osteoblast differentiation, is positive in hypertrophic chondrocytes in the calcified cartilage area [21]. 

These histological findings suggest that OPLL progresses through endochondral ossification.

### 2.2. Histology of Human OLF

Even in OLF samples, the ossification front contains fibrocartilage and calcified cartilage areas, with marked irregularities in the calcified cartilage areas. In addition, Sox9-positive chondrocytes are present around the calcified cartilage area. A convergence of small vessels and osteoblasts is observed around the ossification front. Regularly arranged elastic fibers are seen in non-ossification samples, whereas irregularly arranged and fragmented elastic fibers are seen in OLF samples. TUNEL-positive chondrocytes are found predominantly around the calcified cartilage areas. Hypertrophic chondrocytes in the calcified cartilage areas are negative for Sox9. In contrast, Osterix (Osx)-positive cells are observed; Osx is a transcription factor for osteoblast differentiation [22]. The expression of BMP-2 and vascular endothelial growth factor (VEGF), which has angiogenic effects, is observed in the calcified cartilage and fibrocartilage areas [23].

### 2.3. Hypothesis on the Pathogenesis of OSL

Samples removed at surgery are typically mature ossified lesions, and it is difficult to capture samples representing early stages of ossification. Ligament hypertrophy is considered a precursor to ossification, and reports on posterior longitudinal ligament (PLL) thickened samples show chondrocyte proliferation, vascular invasion, and BMP-2-positive cells [24].

**Figure 1:**
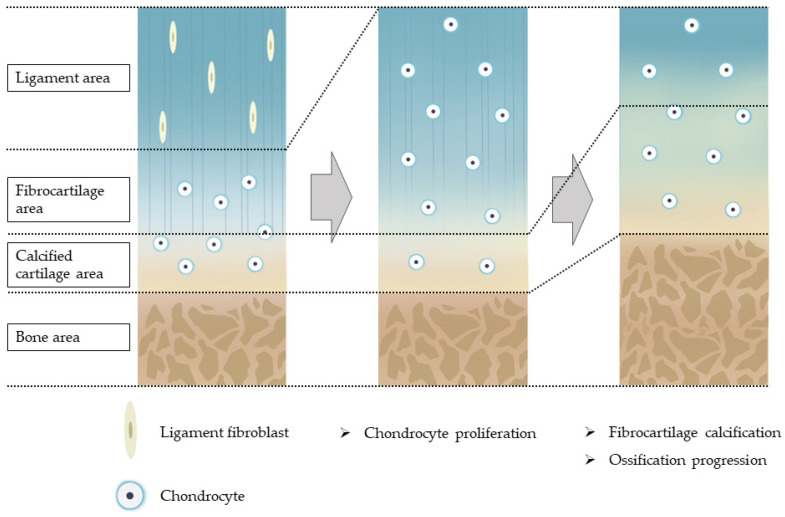
Hypothesis of the pathogenesis of ossification of the spinal ligaments.

In summary, the previous reports suggest that the first step of ossification is the hypertrophy of ligamentous tissues and the proliferation of chondrocytes. This is followed by chondrocyte maturation and calcification, and then ossification proceeds with the entry of osteoblasts and the induction of blood vessels (Figure 1) [25].

## 3. Tissue-Specific Progenitor Cells Involvement

The widely shared view is that mesenchymal stem cells (MSCs) are ubiquitous in mesodermal tissue and are characterized by non-hematopoietic, plastic-adherent, colony-forming unit fibroblasts (CFU-Fs) formation. They also can self-renew, differentiate into bone, cartilage, and fat, and have an in vitro phenotype (expressing CD73, CD90, and CD105 and negative for CD11b, CD14, CD19, CD34, CD45, CD79α, and HLA-DR1) [26,27,28,29,30]. Recently, however, it has been reported that cultured cells, considered ubiquitous MSCs in mesodermal tissues, are progenitor cells with their tissue-specific differentiation potential [31]. Therefore, this article will use the term progenitor cells to avoid confusion.

The involvement of tissue-specific progenitor cells has been reported as the origin of the endochondral ossification of spinal ligaments. Progenitor cells were also isolated from human spinal ligaments. Cells isolated from spinal ligaments were positive for the expression of CD73, CD90, and CD105 and showed low or no indication of CD11b, CD19, CD34, CD45, and HLA-DR. Furthermore, they retain the ability to differentiate into osteoblasts, adipocytes, and chondrocytes, indicating that cells derived from human spinal ligaments have the characteristics of progenitor cells [32].

The localization of progenitor cells in human spinal ligaments is controversial. Several reports suggest that progenitor cells are anatomically and functionally related to the vascular/perivascular niche of the tissue [33]. In non-OLF samples of human spinal ligaments, MSC marker double-positive cells were predominantly present in the perivascular area, with few in the collagen matrix. In contrast, in the OLF sample, in addition to expression in the perivascular area, progenitor cell markers were positive in numerous fibroblast-like cells within the fragmented collagen matrix, suggesting that progenitor cells may migrate from the perivascular area and participate in the repair of micro-damaged areas. Furthermore, perivascular progenitor cell marker-positive cells did not co-localize with CD31, a vascular endothelial cell marker, but with alpha-smooth muscle actin (α-SMA), a pericyte marker, indicating that they were a population of pericytes. Progenitor cell markers were also positive in chondrocytes around calcified cartilage areas. These findings suggest the involvement of tissue-specific progenitor cells in endochondral ossification [34].

Progenitor cells isolated from spinal ligaments of non-OPLL and OPLL patients showed significantly higher osteogenic differentiation potential in OPLL-derived progenitor cells. In addition, osteogenesis-related genes were also significantly higher in OPLL-derived progenitor cells. In contrast, the adipogenic and chondrogenic differentiation and expression of their associated genes were comparable. The increased osteogenic differentiation potential of OPLL-derived progenitor cells suggests that they may be a causative factor in the ossification of spinal ligaments [35].

## 4. ttw Mice

### 4.1. Overview

The tiptoe walking (ttw) mouse is a spontaneous mutant model that develops heterotopic ossification of the spinal ligaments and other parts of the body, beginning in early life. Inheritance is autosomal recessive with complete penetrance. Heterotopic ossification causes contractures in the joints and ligaments of the lower limbs, resulting in a characteristic tiptoe walk [36].

### 4.2. Causative Gene and Function

The phenotype of ttw mice is caused by a point mutation in the ectonucleotide pyrophosphatase/phosphodiesterase 1 (*Enpp1*) gene. The accelerated heterotopic ossification characteristic of ttw mice is due to the dysfunction of ENPP1 caused by cleavage of the gene product, with a loss of more than one-third of the ENPP1 natural protein [37].

ENPP1 is expressed in a wide range of tissues, including cartilage, heart, kidney, parathyroid, and skeletal muscle, and it is highly expressed in vascular smooth muscle cells, osteoblasts, and chondrocytes [38]. 

The function of ENPP1 is to hydrolyze extracellular nucleotide triphosphate to produce either inorganic pyrophosphate (PPi) and adenylate (AMP) or inorganic phosphate (Pi) and adenosine diphosphate (ADP). Pi acts to promote the precipitation of hydroxyapatite (HA) crystals, which are important for calcification, whereas PPi serves as an inhibitor of HA formation and a precursor to Pi. Normal levels of PPi prevent calcification of the soft tissues and blood vessels. Inactivating mutations in the *ENPP1* gene result in reduced levels and loss of the ENPP1 enzyme, which reduces PPi levels in the blood and causes precipitation of calcium, phosphorus, and HA [39,40].

### 4.3. Histology and Ossification Process

The ttw mice have increased nucleus pulposus volume in all discs as a prelude to ossification, which causes anterior and posterior herniation at 6 weeks of age. The cartilage tissue of the fibrous rings is destroyed, and chondrocytes positive for proliferating cell nuclear antigen (PCNA), a marker of cell proliferation, show regenerative proliferation. In addition, the extracellular matrix is positive for chondroitin 4-sulfate proteoglycan, indicating progressive calcification. At 10 weeks, alkaline phosphatase (ALP)-positive osteoblast-like cells are markedly increased within the PLL and posterior intervertebral discs, indicating angiogenesis. At 22 weeks, the number of ALP-positive osteoblast-like mesenchymal cells decreases and bone bridges form between the upper and lower vertebrae [41,42,43]. 

Ossification through the process of endochondral ossification is similar to the human histology, but the preceding herniation of the disc may not be typical in humans.

### 4.4. Clinical Relevance

Homozygous mutations in the gene encoding ENPP1 are associated with a rare autosomal recessive disorder, generalized arterial calcification of infancy (GACI) [44]. Patients with this disease often die in infancy, due to vascular calcification in severe cases. Survivors also usually develop autosomal recessive hypophosphatemic rickets type 2 (ARHR2), in which extravascular calcification occurs; patients with ARHR2 have been reported to develop OSL [45,46,47]. In addition, three patients with heterozygous and compound heterozygous *ENPP1* mutations have been reported to have decreased plasma PPi levels and to have developed severe OSL. This suggests that OSL patients may include those with *ENPP1* mutations [48]. Several promising single nucleotide polymorphisms (SNPs) have been reported in candidate gene association studies, but the effects of these SNPs on ENPP1 production or function remain unclear [49,50].

## 5. ZFR

### 5.1. Overview

Zucker fatty (fa/fa) rat (ZFR) is a mutant caused by a missense mutation in the leptin receptor (*Lepr*) gene on chromosome 5 [51]. Due to this mutation, Leptin receptors are dysfunctional. ZFR has satiety reflex disorder and chronic bulimia, resulting in overeating, severe obesity and many other comorbidities associated with excess adiposity, such as impaired glycemic control and severe hypertriglyceridemia [52]. In ZFR, microscopic small ossifications are observed in the anterior and posterior longitudinal ligaments, and radiologically calcified tissues are observed in the Achilles tendon area, suggesting some predisposition to ossification of the spinal ligaments. Therefore, it may be useful as an animal experimental model to elucidate the precursor state of spinal ligament ossification, the initial stage of ossification, and the predisposing factors for systemic or local ossification.

### 5.2. Causative Gene and Function

Leptin is a secreted protein consisting of 167 amino acids that has been identified as the causative factor in ob/ob mice that exhibit marked obesity [53]. Leptin is produced primarily by adipocytes and it suppresses the appetite via neuropeptides in the hypothalamus. It also acts directly on peripheral tissues to increase energy production, thereby decreasing energy storage in the body. Therefore, leptin is an anti-obesity hormone involved in the control of obesity and weight gain [54].

Separate local and central mechanisms have been suggested for the effects of leptin on the regulation of bone metabolism. Leptin receptors are expressed in many mesenchymal cells in bone and cartilage in the periphery [54,55]. Leptin promotes the osteogenic differentiation of bone marrow stromal cells, osteoblasts and human OLF cells in vitro and increases bone mass after local administration [54,55,56,57].

In contrast, leptin receptors are highly expressed in the hypothalamus, and leptin deficiency increases bone mass via reduced sympathetic function in the hypothalamus [58,59,60]. Therefore, the administration of leptin to the hypothalamus decreases bone mass in mice [59]. However, subsequent reports have shown that leptin gene transfer to the hypothalamus and the administration of high concentrations of leptin conversely increase bone formation [61,62].

Recently, LepR⁺ cells have been reported as a subpopulation of bone marrow stromal cells. LepR⁺ bone marrow stromal cells are around sinusoids and arterioles. LepR⁺ cells arise in the bone marrow during the perinatal period, eventually representing about 0.3% of young adult bone marrow cells. Lineage tracing using *Lepr-cre; tdTomato* mice has shown that LepR⁺ cells differentiate into osteoblasts and adipocytes as they grow. The contribution to chondrocytes was only observed during the healing process of fractures and articular cartilage injuries [63]. The knock out of *Lepr* in bone marrow stromal cells using *Prx1*-*Cre*; *Lepr^fl^*^/*fl*^ mice increased bone formation and decreased adipogenesis. An increase in bone formation was also observed with *Prx1*-*Cre*; *Lepr^fl^*^/*fl*^ mice during the healing process after fracture, suggesting that leptin negatively regulates bone formation. There have been no reports on the localization of LepR+ cells in the spinal ligaments or their function, and further research is expected [64].

Thus, a clear understanding of leptin’s action has not been reached, and further elucidation of its action on bone metabolism is eagerly awaited. In addition to effects of leptin, elevated corticosteroid levels, elevated insulin levels, and hypogonadism may be involved in bone metabolism in ZFR, either alone or in combination with abnormal leptin function.

### 5.3. Histology and Ossification Process

ZFR has been reported to develop spinal ligament ossification with age; in 24-week-old ZFR, the PLL is thickened and collagenous fiber disorganization is observed in the thoracic spine. In addition, endochondral ossification is clearly observed in the PLL. Chondrocytes and osteoblasts can be seen around the calcified cartilage area. In the fibrocartilage area near the ossification area, bone morphogenetic protein receptor 1A (BMPR-1A), one of the BMP receptors, is observed in immature and hypertrophic chondrocytes. Observations over time have identified precursor stages such as the thickening of the ligament and the formation of fibrocartilage areas, suggesting that the disease progresses by endochondral ossification [65].

This is a form of ossification similar to human OSL, in that it is age related and undergoes an endochondral ossification process. However, there have been no reports of OSL in db/db mice with similar *Lepr* gene abnormalities, suggesting that the phenotype of OSL caused by leptin receptor abnormalities may vary greatly among animal species [66]. 

### 5.4. Clinical Relevance

Severe obesity, an early onset of symptoms, and diffuse ossification of spinal ligaments have been shown to be distinct features of patients with myelopathy caused by thoracic OPLL. In addition, thoracic OPLL, unlike cervical OPLL, has been found to have a higher prevalence in women [67].

Serum leptin levels are significantly elevated in female patients with OPLL compared with female controls without OPLL. Serum leptin levels are also significantly higher in female patients whose OPLL extends to the thoracic and/or lumbar spine compared with female patients whose OPLL is limited to the cervical spine [68]. These reports suggest the involvement of leptin and obesity in female patients with early-onset thoracic OPLL.

Genetic association studies have reported on SNPs of the leptin receptor gene, with the G allele of the A861G polymorphism reported to be more frequent in patients with thoracic OPLL [69]. Although there are multiple reports on the phenotype of leptin and leptin receptor abnormalities in humans, no mention has been made of OSL, and the association between clinical leptin gene abnormalities and OSL is unknown [70,71].

## 6. Mechanical Stress-Induced Animal Models

### 6.1. Overview

Several mechanical stress models of the spinal ligaments have been reported and are listed in Table 1.

### 6.2. Details of Each Animal Model

In rats, the caudal spinal ligament is subjected to periodic tensile loading, and cartilage formation is observed near the attachment site of the spinal ligament. Some of the cartilage tissue ossifies. The expression of BMP-2 is observed in the cytoplasm of proliferating chondrocytes [72].

Cyclic tensile loading was applied to the thoracolumbar flavum of rats using a proprietary stress device. MicroCT showed OLF in the 4-, 8-, and 12-week groups, with the amount of ossification increasing over time. Histologically, chondrocytes proliferated in the 4-week-old group and ossification occurred in the 8- and 12-week-old groups [73].

In rabbits, a model was developed in which mechanical stress was focused on the L3-4 segment by posterolateral fixation of L2-3 and L4-5 and resection of the L3-4 paraspinal muscles; mechanical stress induced time-dependent flavum hypertrophy, elastic fiber destruction, and cartilage matrix production. However, no ossification was observed [74,75].

In mice, models have been developed that apply mechanical stress to the flavum by using proprietary devices or by forcing the animal to stand on two legs. However, no cartilage induction or ossification has been reported, even though hypertrophy of the ligaments and degeneration of elastic fibers and increased inflammatory cytokines are observed [76,77].

The presence or absence and morphology of ossification differs depending on the intensity of mechanical loading and the animal species. Reproducibility will be a future issue.

### 6.3. Pathogenesis

#### 6.3.1. Cellular Mechanotransduction

The sensing and response of cells and tissues to mechanical forces and the physical microenvironment are critical to their function and survival. While it has long been recognized that chemical signals regulate cell behavior, it is now well-accepted that mechanical forces play an essential role in regulating cellular function [78,79].

Applying mechanical stimuli to cells activates mechanosensitive ion channels, heterotrimeric G proteins, protein kinases, and other membrane-associated signaling molecules. This triggers downstream signaling cascades that result in force-dependent changes in gene expression [80,81].

Integrins and cadherins are physically bound to the cytoskeletal filament network and linked to nuclear scaffolds, nucleoli, chromatin, and DNA in the nucleus. Mechanical forces applied to the surface activate membrane signaling events and promote structural rearrangements deep within the cytoplasm and nucleus. Additionally, mechanical-based signal propagation is much faster than chemical diffusion or translocation-based signal propagation [82].

It has also been reported that mechanical stimuli to cells promote osteogenic differentiation, mainly in bone marrow stromal cells [83].

#### 6.3.2. Molecular Mechanisms Related to Spinal Ligament Cells

There have been several reports on pathology using spinal ligament-derived cells. Elongation stress on OLF-derived cells increases the expression of osteodifferentiation-related genes as elongation time increases. Cyclic elongation increases the expression of the Indian hedgehog (IHH) signaling pathway, suggesting that cyclic elongation stress may affect the osteogenic differentiation of OLF cells via this pathway [84,85].

In OPLL-derived cells, inflammatory responses associated with nuclear factor-kappa B (NFκB) signaling are activated by mechanical stress. The activation of NFκB signals is dependent on the intercellular junction protein connexin 43 (Cx43). Therefore, treatment with Cx43 short interfering (si)RNA or NFκB inhibitors reduces mechanically induced inflammatory responses and attenuates the mechanically stimulated osteoblast differentiation of OPLL cells [86,87].

#### 6.3.3. Inflammation Involvement

Whether mechanical stress induces inflammation in tendons and ligaments has been the subject of significant controversy, but an increasing number of reports have recently suggested that inflammation is involved [88]. Recent human OLF immunohistochemical analyses have reported the presence of CD68+ macrophages in the vicinity of the ossification area [89]. The presence of cells positive for the inflammatory cytokines interleukin (IL)-6 and IL-1α has also been reported in the flavum of patients with OPLL, suggesting the presence of inflammation in the ossification ligament [90].

The cyclic loading of bovine flexor tendons shows damage to collagen fibers and the extracellular matrix and expression of the inflammatory cytokines cyclooxygenase-2 (COX-2) and IL-6 [91]. The cyclic loading of rat patellar tendons results in increased expression of the collagen-degrading enzyme matrix metalloproteinase-13 (MMP-13) and the inflammatory cytokine IL-1β and microstructural damage in the high-loading group, and only slight changes in the low-loading group [92]. Increased CD14+ monocytes and CD68+ macrophages have been reported in human samples of supraspinatus and Achilles tendonitis [93].

These findings suggest that mechanical stress leads to tendon microtears and inflammation. Heterotopic ossification in rodent limb tendons is thought to be caused by an endochondral ossification process involving four stages: inflammation, chondrogenesis, osteogenesis, and maturation [94,95]. There are well-established models for inducing heterotopic ossification in the tendons of rodent limbs [95,96]. In a mouse model of Achilles tendon injury-induced heterotopic ossification, the depletion of monocyte macrophages by clodronate attenuated the inflammatory response at the wound site, resulting in a decreased amount of heterotopic ossification [97].

In contrast, inflammatory cells have not been identified in the aforementioned models of mechanical stress on the flavum of the rat thoracolumbar transition region or on the rat supraspinatus tendon, suggesting that there may be a process of heterotopic ossification that is not mediated by inflammation [73,98].

### 6.4. Clinical Relevance

In humans, mechanical stress is difficult to quantify, making it challenging to prove its effect on OSL, although several reports strongly suggest an effect of mechanical stress. In case reports of OLF in young Asian baseball pitchers, unilateral predominant ossification occurred in the thoracolumbar region, suggesting that a high degree of asymmetric mechanical loading (i.e., in the pitching motion) may have strongly influenced the ossification [99,100,101].

Epidemiologically, the incidence of OLF peaks at T10-T11 [102,103]. This is because stability due to the ribs and thorax cannot be expected in the thoracolumbar region at T10-L1. Furthermore, the thoracolumbar region is considered to be susceptible to rotational stress, as the lumbar spine is only slightly rotated [104,105]. In a study of 121 dissected cadavers, OLF was observed in 83% of cases in the thoracolumbar region when the intervertebral joints had a thoracic-like configuration and a wide range of rotational motion. In contrast, when the intervertebral joints had a lumbar-like configuration and the range of rotational motion was narrow, the frequency of OLF was 33% [106].

Patients with cervical OPLL who underwent posterior decompression and fixation had a lower rate of ossification progression than those who underwent laminoplasty alone. These reports suggest that the braking of the mechanical load by immobilization may have inhibited the progression of ossification, although the possibility remains that the severity of ossification and cervical alignment may have biased judgment regarding the surgical technique [107,108,109,110,111].

## 7. New Approaches

### 7.1. Epigenetics

In recent years, research in epigenetics has progressed. Extracellular vesicles (EVs) have been proposed to function as a delivery system for mRNA and microRNA (miRNA) and to shuttle proteins to recipient cells; in humans, miRNAs are known to be highly enriched in EVs [112,113].

miRNA-10a was identified as an OPLL-specific miRNA in sequencing data and was shown to promote the osteogenic differentiation of PLL cells in vitro. Furthermore, changes in miRNA-10a expression in PLL cells affected heterotopic bone formation in vivo. The authors reported that miRNA-10a promotes Runx2 function by downregulating the inhibitor of DNA binding 3 (ID3) [114].

The expression of miR-140-5p was downregulated in EVs of OPLL cells. miR-140-5p overexpression EVs delivered to human MSCs inhibited osteogenic differentiation. The authors also reported that miR-140-5p targets insulin-like growth factor 1 receptor (IGF1R), which suppresses the osteogenic differentiation of MSCs by regulating the mammalian target of rapamycin (mTOR) pathway [115].

miR-320e is abundantly expressed in OPLL-derived EVs, and miR-320e promotes the bone differentiation of PLL cells and mesenchymal stem cells. Furthermore, the treatment of ttw mice with OPLL-derived EVs increased OSL, whereas the inhibition of miR-320e attenuated ossification to the same level as in the untreated group. Transforming growth factor-beta-activated kinase-1 (*TAK1*) was identified as a major target gene for miR-320e [116].

### 7.2. Genome-Wide Association Analysis

In 2014, a genome-wide association analysis was performed in a large Japanese population of 1130 OPLL patients and 7135 controls. Six susceptibility loci (6p21.1, 8q23.1, 8q23.3, 12p11.22, 12p12.2, 20p12.3) were identified that met the genomic level of significance. Of the 63 genes present in these genomic regions, five candidate genes (*RSPO2*, *HAO1*, *CCDC91*, *RSPH9*, and *STK38L*) associated with bone and cartilage metabolism were reported [117]. For some of these genes, association with SNPs and functional analysis were performed.

A detailed functional analysis of R-spondin2 (RSPO2) and its SNPs was performed. RSPO2 functions as a ligand for wnt/β-catenin signaling and suppresses early chondrogenic differentiation. A risk SNP (rs374810) is located in the promoter region of *RSPO2* and reduces promoter activity, resulting in the decreased expression of *RSPO2*. In fact, the expression level of *RSPO2* was decreased in human fibroblasts with the risk SNP [118].

RSPO2 is upregulated early in a mouse Achilles tendon injury model that can induce heterotopic ossification. Using single cell (sc) RNA-sequencing analysis, it was shown that the cluster of cells expressing *RSPO2* is simultaneously a cluster of undifferentiated cells labeled with proteoglycan 4 (Prg4). The overexpression of RSPO2 in Prg4-positive cells using the same model suppressed heterotopic ossification via the suppression of chondrogenic differentiation. Thus, RSPO2 may contribute to homeostasis by suppressing the chondrogenic differentiation of tendons and ligaments [119].

## 8. Prospects

Although existing animal models reproduce some of the pathophysiologies of OPLL, challenges remain due to the tenuous clinical relevance, lack of functional elucidation, and difficulty in reproducibility.

Basic research on OSL has made significant progress, including in the identification of disease susceptibility genes and epigenetics. However, there are many challenges in elucidating the pathogenesis of the disease. We hope that the integration of findings from various future research areas, such as genetic factors, environmental factors, and their interactions, and the cellular origin of heterotopic ossification and its mechanisms, will lead to the development of more useful animal models as well as advances in the treatment of OSL.

## Figures and Tables

**Table 1:** Mechanical stress models of the spinal ligaments.

Species	Region	Method	Result	Reference
Rat	Caudal spinal ligament	Cyclic tensile loading	Histological ossification	[72]
Rat	Thoracolumbar flavum	Cyclic tensile loading	Ossification detectable by microCT	[73]
Rabbit	Lumbar flavum	Adjacent vertebral fixation	Cartilage matrix production	[74,75]
Mouse	Lumbar flavum	Bipedal standing	Ligament hypertrophy	[76]
Mouse	Lumbar flavum	Consecutive flexion-extension stress	Ligament hypertrophy	[77]

## Data Availability

Not applicable.
